# A toolkit for *Nannochloropsis oceanica* CCMP1779 enables gene stacking and genetic engineering of the eicosapentaenoic acid pathway for enhanced long‐chain polyunsaturated fatty acid production

**DOI:** 10.1111/pbi.12772

**Published:** 2017-07-13

**Authors:** Eric Poliner, Jane A. Pulman, Krzysztof Zienkiewicz, Kevin Childs, Christoph Benning, Eva M. Farré

**Affiliations:** ^1^ MSU‐DOE Plant Research Laboratory Michigan State University East Lansing MI USA; ^2^ Cell and Molecular Biology Program Michigan State University East Lansing MI USA; ^3^ Department of Plant Biology Michigan State University East Lansing MI USA; ^4^ Department of Plant Biochemistry Albrecht‐von‐Haller‐Institute for Plant Sciences Georg‐August‐University Gottingen Germany; ^5^ Department of Biochemistry and Molecular Biology Michigan State University East Lansing MI USA

**Keywords:** *Nannochloropsis*, LC‐PUFA, eicosapentaenoic acid, multigene expression, gene stacking, genetic engineering toolkit, 2A peptides, bidirectional promoters

## Abstract

*Nannochloropsis oceanica* is an oleaginous microalga rich in ω*3* long‐chain polyunsaturated fatty acids (LC‐PUFAs) content, in the form of eicosapentaenoic acid (EPA). We identified the enzymes involved in LC‐PUFA biosynthesis in *N. oceanica* CCMP1779 and generated multigene expression vectors aiming at increasing LC‐PUFA content *in vivo*. We isolated the cDNAs encoding four fatty acid desaturases (FAD) and determined their function by heterologous expression in *S. cerevisiae*. To increase the expression of multiple fatty acid desaturases in *N. oceanica* CCMP1779, we developed a genetic engineering toolkit that includes an endogenous bidirectional promoter and optimized peptide bond skipping 2A peptides. The toolkit also includes multiple epitopes for tagged fusion protein production and two antibiotic resistance genes. We applied this toolkit, towards building a gene stacking system for *N. oceanica* that consists of two vector series, pNOC‐OX and pNOC‐stacked. These tools for genetic engineering were employed to test the effects of the overproduction of one, two or three desaturase‐encoding cDNAs in *N. oceanica* CCMP1779 and prove the feasibility of gene stacking in this genetically tractable oleaginous microalga. All FAD overexpressing lines had considerable increases in the proportion of LC‐PUFAs, with the overexpression of *Δ12* and *Δ5* FAD encoding sequences leading to an increase in the final ω*3* product, EPA.

## Introduction

Long‐chain polyunsaturated fatty acids (LC‐PUFAs) are hydrocarbon acyl chains (18–22 carbons) containing multiple *cis* double bonds. The double bond closest to the methyl (ω) end of a fatty acid, usually three or six carbons, differentiates LC‐PUFAs into ω*3* or ω*6* classes, respectively. Evidence suggests consuming LC‐PUFAs in adequate amounts with a balanced ratio among LC‐PUFAs is essential for human physical and mental health (Chen *et al*., 2016), while current diets are often low in ω*3* LC‐PUFAs.

Currently, seafood is the major source of ω*3* LC‐PUFAs, particularly EPA (20:5; number of carbons:number of double bonds) and docosahexaenoic acid (DHA, 22:6). However, overfishing, habitat destruction and pollution have reduced wild fish stocks, while increasing human populations drive a record demand for LC‐PUFA nutrients (Betancor *et al*., 2015; Petrie and Singh, 2011). Although fish are rich in ω*3* fatty acids, the majority of LC‐PUFAs acyl chains originate at the base of the food chain in marine microalgae (Doughman *et al*., 2007; Martins *et al*., 2013). Aquaculture of microalgae is an emerging source of LC‐PUFAs for fish farming or direct human consumption, and understanding the biosynthetic pathways and factors that influence LC‐PUFA metabolism in algae will enable increased production from these organisms (Mühlroth *et al*., 2013).

One promising source of LC‐PUFAs is oleaginous microalgae of the *Nannochloropsis* genus, which have a high EPA content (Chini Zittelli *et al*., 1999; Krienitz and Wirth, 2006; Spolaore *et al*., 2006). In *Nannochloropsis* species, EPA is associated with membrane lipids, particularly monogalactosyl diacylglycerol (MDGD) and the betaine lipid, diacylglyceryl‐*N,N,N*‐trimethylhomoserine (DGTS)(Liu *et al*., 2013; Sukenik *et al*., 1989, 1998; Vieler *et al*., 2012). Despite the research interest and economic implications, the EPA biosynthetic pathway in *Nannochloropsis* species is only partially characterized. Gene annotation and ^14^C‐acetate pulse‐chase labelling suggest that EPA biosynthesis occurs via the ω*6* desaturation and elongation pathway and is localized in the endoplasmic reticulum, with the phospholipids, phosphatidylcholine (PC) and phosphatidylethanolamine (PE), as substrates for fatty acid desaturases (FADs) (Kaye *et al*., 2015; Sukenik *et al*., 1998; Vieler *et al*., 2012).

Genome annotation identified putative components of the EPA biosynthetic pathway including *Δ9*,* Δ12*,* Δ6*,* Δ5* and ω*3* FADs, and a *Δ6* fatty acid elongase (FAE) in *N. oceanica* CCMP1779 (Vieler *et al*., 2012). Only the *N. oceanica Δ6* and *Δ12* FADs have been isolated and expressed in *S. cerevisiae* to corroborate their predicted functions (Kaye *et al*., 2015; Ma *et al*., 2011).

Genetic engineering can increase the production of LC‐PUFAs in plants (Hong, 2002; Qi *et al*., 2004; Wu *et al*., 2005), fungi (Okuda *et al*., 2015; Tavares *et al*., 2011; Xue *et al*., 2013) and microalgae (Cook and Hildebrand, 2015; Hamilton *et al*., 2014; Hildebrand *et al*., 2013; Hwangbo *et al*., 2014; Peng *et al*., 2014). For example, the overexpression of the *Δ12* FAD encoding cDNA using a stress‐inducible promoter in *N. oceanica* caused an increase in the 18:2 fatty acid content of PC during normal growth and diversion of 18:2 into triacylglycerol (TAG) following nitrogen deprivation (Kaye *et al*., 2015). Furthermore, ω3 LC‐PUFA production has been introduced into oleaginous yeast and plants by multiple gene expression systems (Ruiz‐Lopez *et al*., 2012, 2014; Xue *et al*., 2013). The effective diversion of metabolism into LC‐PUFA production is likely to require coexpression of multiple genes encoding enzymes that catalyse different desaturation and elongation steps (Cahoon *et al*., 2007; Metz, 2001; Mühlroth *et al*., 2013).

Metabolic engineering of multigene pathways has been developed using a variety of strategies (Halpin, 2005; Naqvi *et al*., 2010). Commonly, each transgene is placed under the regulation of separate promoters and terminators, and single gene expression plasmids are independently introduced into the host or assembled into a larger multigene expression plasmid (Halpin, 2005). However, other strategies exist to express multiple genes. For example, bidirectional promoters allow compact assembly of coregulated gene pairs and have been utilized in a number of transgenic strategies, including in *Nannochloropsis* species (Kilian *et al*., 2011; Moog *et al*., 2015).

An obstacle to compact multigene expression systems in eukaryotes is the requirement of each transgene to be encoded in individual mRNAs. However, introducing short viral 2A peptide coding sequences (Szymczak *et al*., 2004; Szymczak‐Workman *et al*., 2012) prevents the formation of a peptide bond during translation, allowing multiple proteins to be encoded by a single mRNA molecule, which has increased the efficiency of multiple coding sequence expression in eukaryotic cells. Advantages of using 2A peptides include their compact size of 20–60 amino acids (aa), high efficiency in most eukaryotes tested to date, low toxicity and stoichiometric coexpression of linked proteins (de Felipe *et al*., 2003; Kim *et al*., 2011; Sharma *et al*., 2012; Szymczak *et al*., 2004; Szymczak‐Workman *et al*., 2012). However, the efficiency of peptide bond skipping varies depending on the 2A peptide and the host combination; therefore, optimization for the respective host is needed for the approach to become practical.

Using state‐of‐the‐art technology, we developed vectors that combine a highly active unidirectional promoter (EF) or a bidirectional promoter (Ribi) with a variety of reporter proteins and epitope tags for optimized transgene expression in *N. oceanica* CCMP1779. Moreover, we optimized a viral 2A peptide for polycistronic expression to generate a multigene expression system for this oleaginous microalga. This vector toolkit was successfully used to manipulate the EPA biosynthesis pathway in *N. oceanica* CCMP1779.

## Results

### Genes encoding enzymes of the eicosapentaenoic acid biosynthesis pathway are coexpressed

**Figure 1:**
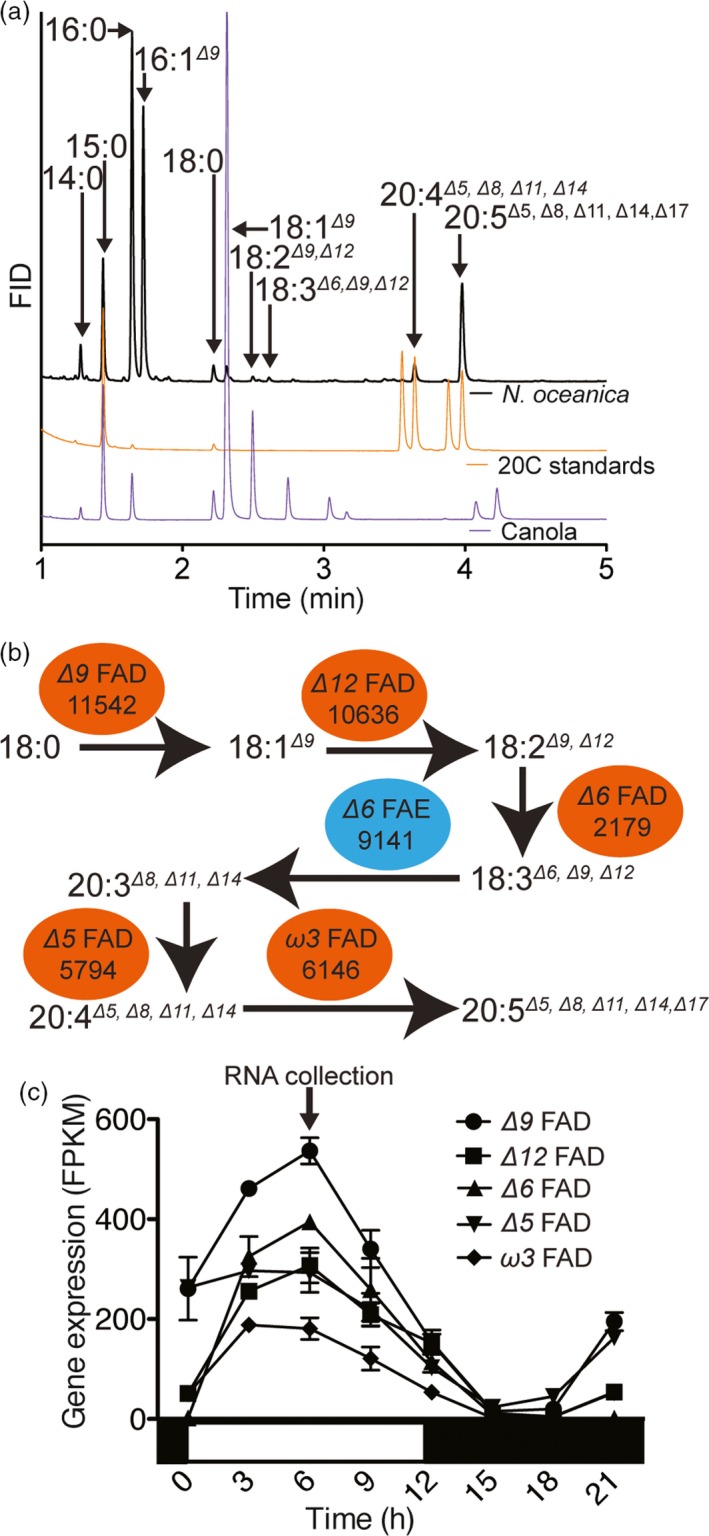
EPA biosynthetic pathway identification in *N. oceanica* CCMP1779. (a) Identification of the fatty acid species by comparison with canola oil and 20C LC‐PUFAs standards (20:3^*Δ8,Δ11,Δ14*^, di‐homo gamma linolenic acid; 20:4^*Δ5,Δ8,Δ11,Δ14*^, ω*6* eicosatetraenoic acid; 20:4^*Δ8,Δ11,Δ14,Δ17*^, ω*3* eicosatetraenoic acid; and 20:5^*Δ5,Δ8,Δ11,Δ14,Δ17*^, eicosapentaenoic acid). (b) The EPA biosynthetic pathway includes five desaturases (red ovals, FADs) and an elongase (blue oval, FAE). Gene IDs are shortened from the *NannoCCMP1779_#* format. (c) Expression of the EPA biosynthetic pathway encoding genes during a light:dark cycle. The arrow indicates the time of cell harvest for cDNA isolation of EPA biosynthetic pathway genes. Gene expression was calculated using a previous experiment (Poliner *et al*., 2015) and the corrected gene annotation. Values are average ± range of two independent cultures.

The identification of ω*6* intermediates (20:4^*Δ5,Δ8,Δ11,Δ14*^) (Figure 1a) points to the presence of an ω*6* pathway for EPA biosynthesis in *N. oceanica* CCMP1779 (Figure 1b). We isolated the cDNAs for the five FADs and a putative *Δ6* fatty acid elongase (FAE) proposed to be involved in LC‐PUFA biosynthesis in *N. oceanica* CCMP1779 (Figure 1b). These cDNA sequences served to update the gene models of the *Δ9*,* Δ12*,* Δ6*,* Δ5*, ω*3* FADs and *Δ6* FAE. Their updated cDNA sequences were deposited at NCBI with the accession numbers KY214449, KY214450, KY214451, KY214453, KY214454 and KY214452, respectively. Using the corrected gene models, we observed that the FAD genes are highly coexpressed under light/dark conditions with a maximum expression 6 h after dawn (Figure 1c).

**Figure 2:**
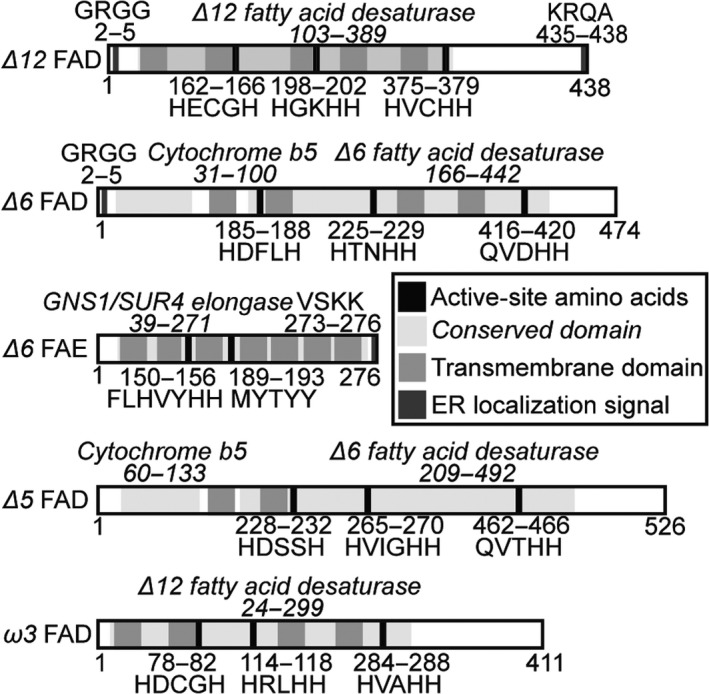
Computational annotation of protein sequences of isolated EPA biosynthetic genes. Protein sequences were generated based upon isolated cDNA sequences. Active‐site amino acids and conserved domains identified using conserved domain BLAST. ER localization signals identified with HECTAR. Transmembrane domains predicted using TMHMM2.

Computational tools and manual examination were used to predict functional domains, subcellular localization and transmembrane sequences in these proteins in support of their initial functional annotations (Figure 2). The FADs contain typical fatty acid desaturase domains, in particular the crucial three histidine boxes for coordinating a diiron centre in the active site (Broun, 1998; López Alonso *et al*., 2003). Moreover, the *Δ6* and *Δ5* FADs contain a cytochrome b5 domain that donates electrons for desaturation as observed for front‐end desaturases of eukaryotic origin, as well as glutamine substitutions in the third histidine box characteristic of front‐end desaturases (Domergue *et al*., 2002; Hashimoto *et al*., 2008; Meesapyodsuk and Qiu, 2012).

The *Δ6* FAE contains an ELO family elongase domain, which is involved in very‐long‐chain fatty acid elongation and sphingolipid formation (Oh *et al*., 1997; Tvrdik *et al*., 2000). Further evidence of a possible elongase function is provided by the presence of FLHXYHH and MYSYY motifs characteristic of *Δ6* and *Δ5* fatty acid elongases; the first is positioned with an upstream glutamine characteristic of PUFA elongases, and the latter is typically found in microalga *Δ6* and *Δ5* fatty acid elongases (Hashimoto *et al*., 2008; Jiang *et al*., 2014; Yu *et al*., 2012) (Figure 2).

All the *N. oceanica* CCMP1779 EPA biosynthesis proteins are predicted to contain membrane‐spanning domains by TMHMM2 (Moller *et al*., 2001). The stramenopile‐specific subcellular localization algorithm HECTAR (Gschloessl *et al*., 2008) predicted endoplasmic reticulum localization signals for the *Δ12* and *Δ6* FADs, and *Δ6* FAE (Figure 2).

### *N. oceanica* CCMP1779 FADs catalyse the production of LC‐PUFAs in yeast

**Figure 3:**
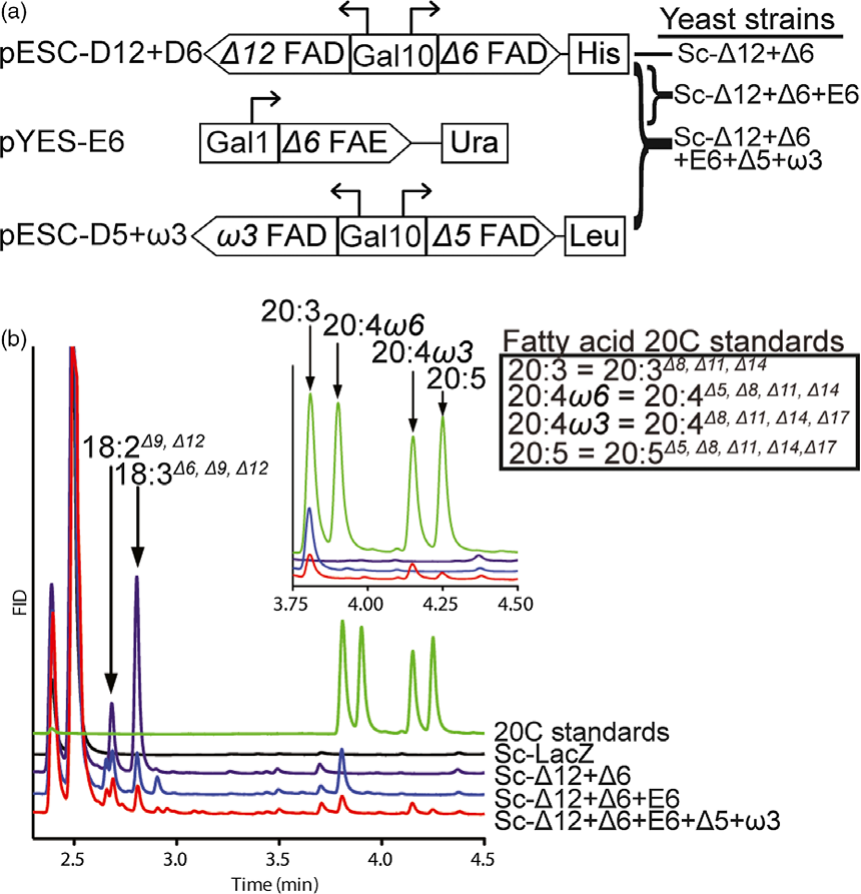
Galactose‐inducible expression of the EPA pathway genes in *S. cerevisiae*. (a) The stacking strategy for EPA production in *S. cerevisiae* used three plasmids, containing different auxotrophic markers. Dual expression of FADs (pESC‐D12 + D6, and pESC‐D5 + W3) was achieved by a bidirectional Gal10 promoter, and expression of the FAE was under control of the Gal1 promoter (pYES‐E6). (Co)transformation of vectors yielded *S. cerevisiae* strains Sc‐Δ12 + Δ6, Sc‐Δ12 + Δ6 + E6 and Sc‐Δ12 + Δ6 + E6 + Δ5 + ω3. (b) Representative GC‐FID fatty acid profiles of Sc‐LacZ‐negative control and FAD expressing yeast strains 48 h postgalactose induction.

To test the biological activity of the putative FADs and FAE from *N. oceanica* CCMP1779, the pathway was reconstituted in the heterologous host *Saccharomyces cerevisiae* using a gene stacking strategy (Figure 3a). *S. cerevisiae* contains a single *Δ9* FAD which produces 16:1^*Δ9*^ and 18:1^*Δ9*^ (Stukey *et al*., 1990). Therefore, to generate EPA from the endogenous 18:1^*Δ9*^, it is necessary to introduce four additional desaturases and an elongase. Towards this end, we coexpressed cDNAs encoding the *Δ12*,* Δ6* and *Δ5*, ω*3* FADs and the *Δ6* FAE under the control of galactose‐inducible promoters in the *S. cerevisiae* strain InvSc1 (Figure 3a).

The presence of these constructs in the absence of galactose induction or the introduction of a *LacZ* expression control vector (Sc‐LacZ strain) did not alter the cellular fatty acid composition of *S. cerevisiae* (Figure 3b, Figure S1a, Table S1). Induction of the *Δ12* and *Δ6* FADs led to the production of 18:2^*Δ9,Δ12*^ (1.8% of total fatty acids) and 18:3^*Δ6,Δ9,Δ12*^ (4.1%) in the Sc‐Δ12 + Δ6 strain (Figure 3b). The putative *Δ6* FAE was introduced in combination with *Δ12* and *Δ6* FADs resulting in elongation of 18:3^*Δ6,Δ9,Δ12*^ to 20:3^*Δ8,Δ11,Δ14*^ (di‐homo gamma linolenic acid, DGLA) in the Sc‐Δ12 + Δ6 + E6 strain (Figure 3b). Finally, coexpression of the *Δ12*,* Δ6*,* Δ5*, ω*3* FADs and the *Δ6* FAE in the Sc‐Δ12 + Δ6 + E6 + Δ5 + ω3 strain resulted in the formation of EPA (20:5^*Δ5,Δ8,Δ11,Δ14,Δ17*^) (0.1%) (Figure 3b, Table S1). Reconstruction of this LC‐PUFA pathway in *S. cerevisiae* documented the combined functionality of the proteins as predicted from their respective sequences.

Interestingly, the 20:4 fatty acid produced by Sc‐Δ12 + Δ6 + E6 + Δ5 + ω3, 20:4^*Δ8,Δ11,Δ14,Δ17*^ differed from the major 20:4^*Δ5,Δ8,Δ11,Δ14*^ found in *Nannochloropsis* (Vieler *et al*., 2012) (Figure 1a, Figure 3b). This difference could be due to the ω*3* FAD acting on 20:3, preceding the *Δ5* reaction when present in *S. cerevisiae*. To test this hypothesis, we expressed the *Δ5* and ω*3* FADs individually and in combination in yeast to generate the strains Sc‐Δ5 + ω3, Sc‐Δ5 and Sc‐ω3. When Sc‐Δ5 + ω3 was grown in the presence of exogenous 20:3^*Δ8,Δ11,Δ14*^, this strain was able to produce 20:4^*Δ8,Δ11,Δ14,Δ17*^ and EPA (20:5^*Δ5,Δ8,Δ11,Δ14,Δ17*^) establishing the activity of the final two desaturases (Figure S1a, Table S1). To further examine the substrate specificity of the *Δ5* and ω*3* FADs, yeast expressing the individual FADs were supplied 20:3^*Δ8,Δ11,Δ14*^ and either 20:4^*Δ8,Δ11,Δ14,Δ17*^ or 20:4^*Δ5,Δ8,Δ11,Δ14*^, individually or in combination (Figure S1b‐d, Table S1). When supplied with 20:3^*Δ8,Δ11,Δ14*^, Sc‐Δ5 produced 20:4^*Δ5,Δ8,Δ11,Δ14*^ and Sc‐ω3 produced 20:4^*Δ8,Δ11,Δ14,Δ17*^ (Figure S1b, Table S1). Supplying Sc‐Δ5 and Sc‐ω3 with 20:4^*Δ8,Δ11,Δ14,Δ17*^and 20:4^*Δ5,Δ8,Δ11,Δ14*^ *,* respectively, resulted in the production of EPA (20:5^*Δ5,Δ8,Δ11,Δ14,Δ17*^) (Figure S1c, Table S1). Sc‐Δ5 supplied with 20:3^*Δ8,Δ11,Δ14*^ and 20:4^*Δ8,Δ11,Δ14,Δ17*^ resulted in the respective products 20:4^*Δ5,Δ8,Δ11,Δ14*^ and EPA (20:5^*Δ5,Δ8,Δ11,Δ14,Δ17*^) (Figure S1d, Table S1). Supplying Sc‐ω3 with 20:3^*Δ8,Δ11,Δ14*^ and 20:4^*Δ5,Δ8,Δ11,Δ14*^ resulted in the production of 20:4^*Δ8,Δ11,Δ14,Δ17*^ and EPA (20:5^*Δ5,Δ8,Δ11,Δ14,Δ17*^) (Figure S1d, Table S1). These experiments revealed that the *Δ5* FAD can accept either ω*3* (20:4^*Δ8,Δ11,Δ14,Δ17*^) or ω*6* (20:3^*Δ8,Δ11,Δ14*^) substrates, and the ω*3* FAD can accept either 20:3^*Δ8,Δ11,Δ14*^ or 20:4^*Δ5,Δ8,Δ11,Δ14*^ substrates when produced in yeast.

### A vector toolkit for multigene expression in *Nannochloropsis* species

**Figure 4:**
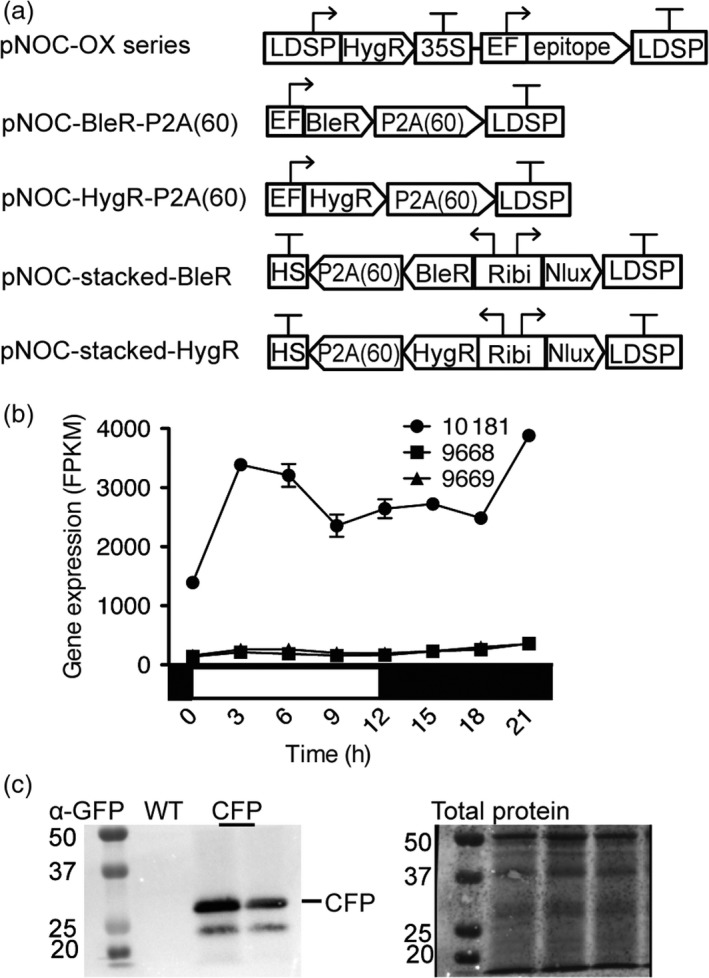
Assembly of native promoters, terminators and a range of reporters to generate a transgenic expression toolkit for *N. oceanica* CCMP1779. (a) The pNOC‐OX vector series contains a series of reporters under the control the EF promoter with the LDSP terminator, and a hygromycin resistance gene under the control of the LDSP promoter and 35S terminator. Epitopes include the cyan fluorescent protein (CFP), yellow fluorescent protein (YFP), green fluorescent protein (GFP) and NanoLuciferase (Nlux), and hemagglutinin peptide (HA) encoding sequences. P2A(60) is an extended 2A peptide coding sequence placed 3′ of the zeocin resistance gene (BleR) or hygromycin resistance gene (HygR) for bicistronic expression by ribosomal skipping. The pNOC‐stacked vectors utilize a bidirectional promoter (Ribi) for coexpression of a reporter and resistance gene with 2A peptide coding sequence and multicloning site followed by a heat‐shock terminator. (b) RNA expression of endogenous genes corresponding to the promoters, *NannoCCMP1779_10181* (EFpro), and the gene pair *NannoCCMP1779_9669* and *NannoCCMP1779_9669* (Ribi promoter) under light:dark cycles (data from (Poliner *et al*., 2015)). (c) Transgenic protein confirmation by immunoblot of pNOC‐OX‐CFP transformants detected with α‐GFP. Total protein was stained using the dye DB71.

To facilitate the coexpression of multiple coding sequences and characterization of the respective enzymes, we generated a set of vectors to overexpress multiple FADs in *N. oceanica* (Figure 4a). For single transcript expression vectors, we chose the elongation factor promoter (EFpro)(*NannoCCMP1779_10181*) due to its high constitutive activity during light:dark cycles (Figure 4b). We generated a vector series (pNOC‐OX) encoding a variety of reporter proteins and epitope tags, including hemagglutinin (HA), green fluorescent protein (eGFP), cyan fluorescent protein (cerulean), yellow fluorescent protein (venus) and the ultra‐bright codon optimized (Table S2) NanoLuciferase (Nlux) (Hall *et al*., 2012), flanked by glycine‐serine‐glycine encoded linkers and a set of compatible restriction sites (AscI/HpaI and MluI/NruI) which enable translational fusion of the epitope tags to either the C or N terminus of the targeted protein (Figure 4a).

To identify candidate bidirectional promoters in *N. oceanica*, a custom python script (Data S1) was used to find diverging gene pairs that coexpressed (Table S3). We selected the intergenic region between two ribosomal subunits as a promising candidate bidirectional promoter (Ribi) due to a high degree of coexpression and moderate expression levels throughout the light:dark cycle of the respective gene pair (*NannoCCMP1779_9669, NannoCCMP1779_9668*) (Figure 4b). The Ribi promoter (after modification to remove MluI and NruI recognition sites, Figure S2) was assembled with the best performing selection marker P2A cassette coding sequences (BleR‐P2A(60) and HygR‐P2A(60)) and the toolkit epitope tag coding sequences to generate the pNOC‐stacked vector series (Figure 4a).

To test the newly developed vectors, we transformed *N. oceanica* CCMP1779 with pNOC‐OX‐CFP, pNOC‐OX‐Nlux and pNOC‐stacked‐Nlux. Production of CFP and Nlux was confirmed in selected strains by immunoblotting with α‐GFP and α‐HA antibodies, respectively (Figure 4c, Figure S3a). To assess the activity of the selected promoters, transformants of *N. oceanica* with pNOC‐OX‐Nlux and pNOC‐stacked‐Nlux were screened for their luminescence signals. To quantitatively compare Nlux reporter signal from each promoter, luminescence from an equal number of cells of pNOC‐OX‐Nlux and pNOC‐stacked‐Nlux transformants was measured. Reflecting the higher activity level of EFpro than Ribi, the luminescence of Nlux in pNOC‐OX‐NLux was greater than in pNOC‐stacked‐Nlux lines (Figure S3b).

**Figure 5:**
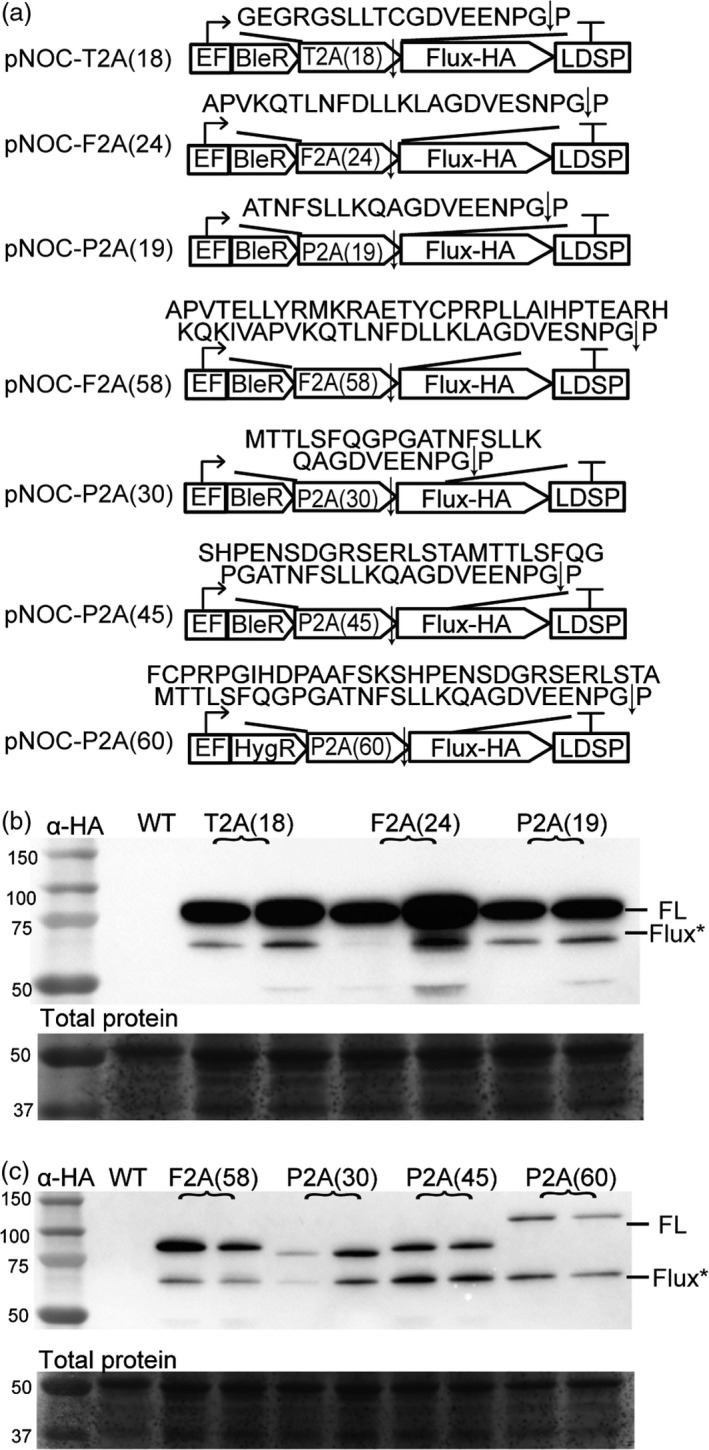
Optimization of 2A peptide ribosomal skipping efficiency in *N. oceanica* CCMP1779. (a) Schematic of pNOC‐2A vector series containing a zeocin resistance coding sequence (BleR) followed by a 2A peptide coding sequence and a multicloning site. Numbers in parentheses correspond to numbers of amino acids of the 2A peptide incorporated, and the encoded amino acid sequence is above (arrow indicates skipping site). For assessing function in *N. oceanica* CCMP1779, a coding sequence for firefly luciferase (Flux) with a C‐terminal HA tag was inserted downstream of the 2A peptide. (b‐c) Immunoblotting with α‐HA antibody of lines transformed with the pNOC‐2A vector series expressing full‐length BleR‐2A‐Flux (FL) and Flux (Flux*). Total protein stained using DB71.

Viral‐derived 2A peptides are used for polycistronic expression of multiple transgenes in eukaryotes and are widely used to tie resistance markers to the production of target proteins. We first determined the ribosomal skipping efficiency of the three most commonly used 2A peptides of ~20 aa, designated 2A peptide (amino acid length). The F2A(24), T2A(18) and P2A(19) coding sequences were appended to the zeocin/bleomycin (BleR) and/or hygromycin (HygR) resistance marker genes followed by insertion of the HA‐tagged firefly luciferase (Flux) coding sequence (Figure 5a). Introduction of these constructs into *N. oceanica* CCMP1779 resulted in zeocin‐ or hygromycin‐resistant colonies, of which those with high luciferase activity were selected for further study. Immunoblotting detected full‐length BleR‐2A‐Flux protein, and only small amounts of released Flux in the first round of screening (Figure 5b, Figure S4). Ribosomal skipping efficiency was less than 10% for F2A, and T2A sequences, while P2A had an efficiency of 10‐30% (Figure S4c). In order to increase ribosomal skipping efficiency, the N‐terminal F2A peptide‐encoding sequence was extended to 58 aa, and the P2A sequence to 30 aa, 45 aa and 60 aa. These changes enhanced the ribosomal skipping efficiency for F2A(58) to ~20%, for P2A(30) to ~40%, for P2A(45) to ~50% and for P2A(60) to >50% (Figure 5b, Figure S4). Based on these results, the extended P2A sequence was selected as the most promising 2A peptide for use in *N. oceanica* CCMP1779.

### Overexpression of EPA biosynthesis genes in *N. oceanica* CCMP1779

**Figure 6:**
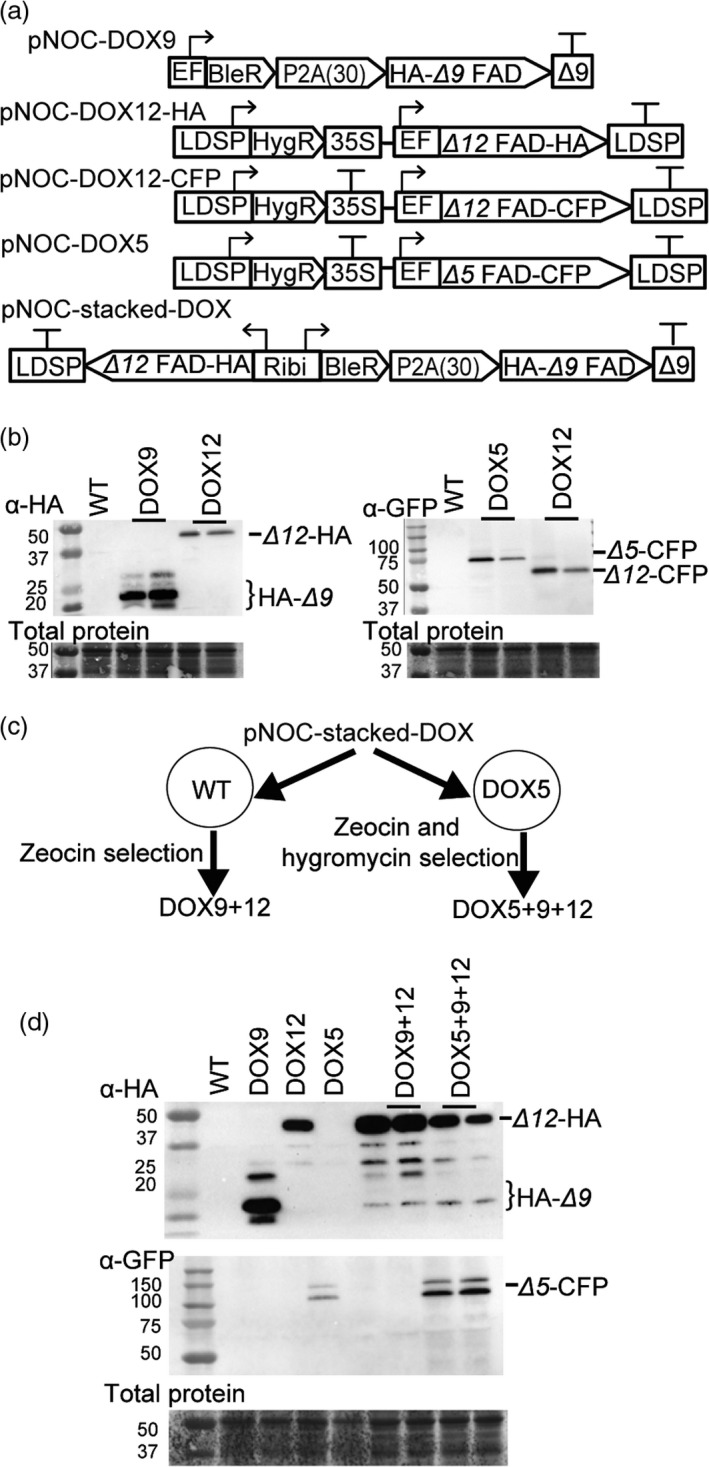
Vectors for FAD overexpression in *N. oceanica* CCMP1779 FADs. (a) Schematics of pNOC‐DOX vectors for overexpression of *N. oceanica* FADs. The *Δ9* FAD coding sequence was expressed 3′ of the BleR‐P2A(30) coding cassette in the vector pNOC‐DOX9. Expression of *Δ5* or *Δ12* FAD coding sequences by a pNOC‐OX vector with a CFP or HA epitope tag. The stacking vector (pNOC‐stacked‐DOX) uses the Ribi promoter to coexpress the coding sequences for BleR‐P2A(30)‐*Δ9* FAD, and *Δ12* FAD. (b) Immunoblotting *N. oceanica* single desaturase DOX lines using α‐HA or α‐GFP antibodies. Total protein was stained using DB71. (c) Gene stacking strategy for *N. oceanica* by use of bicistronic pNOC‐stacked‐DOX and sequential introduction of vectors with different selection markers. (d) Immunoblotting of stacked DOX9 + 12 and DOX5 + 9 + 12 lines using α‐HA or α‐GFP antibodies. Total protein was stained using the dye DB71.

We first generated lines expressing the *Δ9*,* Δ12* and *Δ5* FADs under the control of the EF promoter in *N. oceanica* CCMP1779 (designated DOX9, DOX12 and DOX5 lines, respectively) (Figure 6a). The *Δ9* FAD coding sequence was cloned into pNOC‐P2A(30) (Figure 5) to generate the pNOC‐DOX9 vector. The *Δ12* and *Δ5* FAD coding sequences were placed in overexpression vectors with C‐terminal epitope tags (pNOC‐DOX12‐HA, pNOC‐DOX12‐CFP, pNOC‐DOX5). Lines with changes in their fatty acid profiles were selected for further studies. The DOX12 and DOX5 lines produced appropriately sized proteins (Figure 6b). However, we did not detect full‐length HA‐*Δ9* FAD in DOX9 lines but only several small molecular weight polypeptide products (Figure 6b), which could be due to degradation of the tagged protein. Confocal microscopy of DOX5 and DOX12 lines revealed that the desaturase CFP fusion proteins have an ER subcellular location, as indicated by overlap with an ER‐specific marker dye, supporting the protein annotation and predicted location (Figure S5).

To test whether overproduction of more than one FAD would improve LC‐PUFA accumulation in *N. oceanica* CCMP1779, we transformed wild‐type and the DOX5 strain B3 with a desaturase stacking vector, containing the HA‐*Δ9* FAD and *Δ12* FAD‐HA coding regions under the control of the Ribi bidirectional promoter (pNOC‐stacked‐DOX9 + 12) (Figure 6a,c). DOX9 + 12 and DOX5 + 9 + 12 lines with changes in their fatty acid profile were selected for further analyses. Immunoblotting of selected lines detected full‐length *Δ12* FAD‐HA and HA‐*Δ9* FAD peptides in all lines, and *Δ5* FAD‐CFP in the triple FAD overexpression lines (Figure 6d).

**Figure 7:**
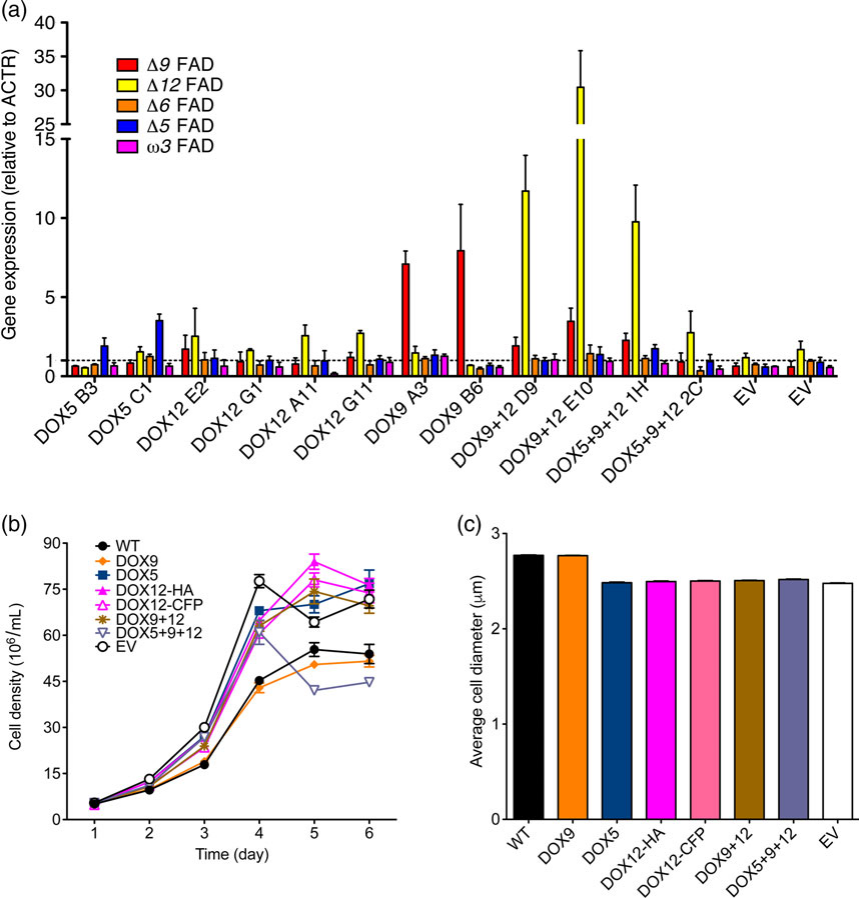
Desaturase overproduction alters the fatty acid profile of *N. oceanica* CCMP1779. (a) Gene expression of FADs measured by qPCR using the *ACTR* gene as control (average ± SEM of 3 independent cultures). (b) Cell growth of DOX lines during 5 days under constant light (average ± SEM of four independent cultures). (c) Cell diameter of DOX lines (average ± SEM of four independent cultures). WT, wild type; EV, empty vector control.

To determine the level of overexpression, mRNA levels were quantified by qPCR (Figure 7a). Lines transformed with the pNOC‐OX vectors (Figure 4a) resulted in expression up to 3.5‐fold higher than the wild type, while DOX9 overexpression using pNOC‐P2A(30) led to ~eightfold increase in mRNA. In the DOX9 + 12 lines, the *Δ12* FAD mRNA content was 10–30 times that of the wild type and displayed large differences between the lines, while the *Δ9* FAD expression was increased two‐ to fourfold.

### Increase in LC‐PUFA content in FAD overexpressing lines

Fatty acid profiling of wild‐type, empty vector controls (EV) and DOX lines showed that an increase in FAD production altered fatty acid proportions (Table 1). While EV controls did not cause alterations to the fatty acid profile compared to the wild type, the overexpression of *Δ9* FAD led to a small increase in the mol per cent of its product 18:1^*Δ9*^ and the overexpression of the *Δ12* or *Δ5* FADs resulted in a higher LC‐PUFA fraction. We did not observe a further increase in LC‐PUFAs in the stacking lines, DOX9 + 12 and DOX5 + 9 + 12, and these lines had a similar ~25% increase in EPA (20:5^*Δ5,Δ8,Δ11,Δ14,Δ17*^) and 35% increase in LC‐PUFAs mol ratio as the single FAD overexpressing lines (Table 1).

**Table 1:** Fatty acid mole per cent of *N. oceanica CCMP1779* strains

	Mol per cent								
Lines	14:0	16:0	16:1	18:0	18:1	18:2	20:4	20:5	LC‐PUFA
WT1	4.13 ± 0.35	37.83 ± 1.55	32.34 ± 0.72	1.77 ± 0.33	1.94 ± 0.10	0.90 ± 0.16	3.66 ± 0.42	17.36 ± 1.08	22.01 ± 1.61
WT2	3.84 ± 0.32	38.28 ± 1.66	33.40 ± 0.55	1.49 ± 0.14	1.98 ± 0.07	0.80 ± 0.11	3.41 ± 0.32	16.80 ± 1.23	21.01 ± 1.59
DOX5 B3	6.17 ± 0.39	**34.35 **±** 1.49**	**27.87 **±** 0.14**	1.41 ± 0.20	1.43 ± 0.32	1.62 ± 0.18	5.83 ± 0.22	**21.27 **±** 1.27**	**28.79 **±** 1.69**
DOX5 C1	6.45 ± 0.22	**34.33 **±** 1.36**	**26.43 **±** 0.51**	1.33 ± 0.24	1.27 ± 0.15	1.24 ± 0.12	5.21 ± 0.11	**23.52 **±** 1.60**	**30.21 **±** 1.86**
DOX12 A11	6.02 ± 0.21	35.79 ± 1.51	**26.43 **±** 0.21**	1.19 ± 0.18	0.82 ± 0.22	1.27 ± 0.25	5.27 ± 0.24	**23.04 **±** 1.11**	**29.76 **±** 1.62**
DOX12 G11	6.30 ± 0.11	35.93 ± 1.55	**27.03 **±** 0.44**	1.23 ± 0.09	1.02 ± 0.12	1.38 ± 0.18	5.53 ± 0.21	**21.27 **±** 1.28**	**28.50 **±** 1.73**
DOX9 A3	3.49 ± 0.04	34.85 ± 0.91	33.49 ± 0.35	1.22 ± 0.13	3.08 ± 0.18	1.30 ± 0.12	4.61 ± 0.18	17.85 ± 0.92	**23.87 **±** 1.21**
DOX9 B6	3.42 ± 0.09	**33.65 **±** 1.02**	35.25 ± 0.09	1.21 ± 0.13	3.77 ± 0.32	1.30 ± 0.06	4.97 ± 0.20	16.43 ± 1.11	**22.70 **±** 1.30**
DOX9 + 12 9D	6.25 ± 0.30	34.88 ± 0.69	**26.76 **±** 0.34**	1.13 ± 0.18	1.70 ± 0.17	1.93 ± 0.15	5.82 ± 0.13	**21.53 **±** 0.62**	**29.29 **±** 0.76**
DOX9 + 12 10E	6.55 ± 0.22	35.72 ± 0.96	**26.39 **±** 0.18**	0.98 ± 0.34	0.89 ± 0.32	2.05 ± 0.23	5.93 ± 0.20	**21.51 **±** 0.96**	**29.48 **±** 1.33**
DOX5 + 9 + 12 1H	6.25 ± 0.31	34.91 ± 1.46	**27.61 **±** 0.49**	1.19 ± 0.23	1.35 ± 0.13	1.64 ± 0.24	5.83 ± 0.29	**21.11 **±** 1.03**	**28.71 **±** 1.63**
DOX5 + 9 + 12 2C	6.66 ± 0.32	**33.43 **±** 1.40**	**27.04 **±** 0.13**	1.21 ± 0.25	1.51 ± 0.16	2.18 ± 0.27	6.37 ± 0.31	**21.47 **±** 0.92**	**30.15 **±** 1.46**
EV F1	3.94 ± 0.13	38.09 ± 1.21	35.69 ± 0.21	1.46 ± 0.12	2.06 ± 0.05	0.59 ± 0.06	2.93 ± 0.19	15.24 ± 0.90	18.76 ± 1.15
EV G2	3.62 ± 0.06	38.24 ± 1.58	35.81 ± 0.26	1.59 ± 0.15	1.98 ± 0.05	0.41 ± 0.10	2.79 ± 0.22	15.57 ± 1.15	18.76 ± 1.45

Fatty acid mole percentage was determined by normalizing the mole content of each fatty acid class against the total moles of all fatty acids. Average ± SEM of four independent cultures. Bold indicates a significant difference with both empty vector (EV) controls (ANOVA and Bonferroni's post hoc test, *P* < 0.05). 16:1^*Δ9*^ *;* 18:1^*Δ9*^ *;* 18:2^*Δ9,Δ12*^ *;* 20:4^*Δ5,Δ8,Δ11,Δ14*^ *;* 20:5^*Δ5,Δ8,Δ11,Δ14,Δ17*^.

To further assess the impact of FAD overproduction, we measured cell growth over several days (Figure 7b). The growth rate of EV, DOX5, DOX12, DOX9 + 12 and DOX5 + 9 + 12 lines, but not DOX9, was increased with respect to the wild type (Figure S6). This effect could be related to the decreased average cell sizes of these lines (Figure 7c). Total cellular fatty acid content per cell was likewise decreased in DOX5, DOX12, DOX9 + 12 and DOX5 + 9 + 12 lines, while EV and DOX9 lines were unaffected (Table 2).

**Table 2:** Cellular fatty acid content of *N. oceanica* CCMP1779 strains

	ng 10^−6^ cells								
Lines	14:0	16:0	16:1	18:0	18:1	18:2	20:4	20:5	Total
WT1	14.9 ± 0.8	157.2 ± 18.4	133.1 ± 13.2	8.4 ± 2.3	8.8 ± 1.0	3.9 ± 0.4	17.4 ± 0.8	83.7 ± 5.0	427.8 ± 35.4
WT2	15.8 ± 0.7	180.4 ± 20.0	155.4 ± 13.0	7.9 ± 1.3	10.3 ± 1.1	4.0 ± 0.4	18.6 ± 1.0	91.9 ± 6.0	484.3 ± 36.4
DOX5 B3	17.7 ± 2.3	**110.3 **±** 10.7**	**88.5 **±** 7.4**	5.0 ± 0.8	5.1 ± 1.4	5.6 ± 0.7	22.1 ± 1.7	79.6 ± 5.7	**334.0 **±** 26.1**
DOX5 C1	18.8 ± 1.3	**112.7 **±** 8.1**	**86.0 **±** 4.8**	4.8 ± 0.9	4.5 ± 0.5	4.4 ± 0.5	20.3 ± 1.0	91.4 ± 9.5	**344.0 **±** 20.8**
DOX12 A11	18.5 ± 0.8	**123.9 **±** 7.1**	**90.7 **±** 2.6**	4.5 ± 0.7	3.2 ± 0.9	4.8 ± 0.9	21.6 ± 0.9	93.7 ± 3.3	**361.6 **±** 8.6**
DOX12 G11	16.0 ± 0.9	**103.6 **±** 10.6**	**76.8 **±** 5.4**	4.0 ± 0.5	3.3 ± 0.6	4.2 ± 0.4	18.7 ± 0.7	71.1 ± 2.8	**298.7 **±** 19.7**
DOX9 A3	18.0 ± 1.6	202.0 ± 20.7	191.6 ± 15.3	8.0 ± 1.4	19.8 ± 2.7	8.1 ± 0.9	31.5 ± 2.5	121.2 ± 9.6	601.0 ± 52.0
DOX9 B6	16.0 ± 2.2	176.1 ± 24.6	182.2 ± 23.0	7.3 ± 1.8	21.8 ± 3.5	7.3 ± 0.8	30.3 ± 2.5	100.2 ± 11.9	541.2 ± 68.2
DOX9 + 12 9D	16.6 ± 1.3	**104.1 **±** 7.0**	**79.2 **±** 4.7**	3.7 ± 0.7	5.7 ± 0.8	6.3 ± 0.6	20.6 ± 1.2	75.5 ± 3.7	**311.7 **±** 17.7**
DOX9 + 12 10E	17.1 ± 1.9	**105.6 **±** 12.6**	**77.0 **±** 7.9**	3.4 ± 1.2	3.1 ± 1.2	6.4 ± 0.3	20.5 ± 1.4	73.7 ± 4.8	**306.9 **±** 29.8**
DOX5 + 9 + 12 1 h	16.8 ± 01.0	**107.1 **±** 12.5**	**83.2 **±** 6.5**	4.2 ± 1.2	4.6 ± 0.8	5.4 ± 0.7	20.9 ± 1.5	74.9 ± 3.4	**317.4 **±** 25.6**
DOX5 + 9 + 12 2C	18.7 ± 1.3	**97.9 **±** 11.6**	**81.8 **±** 8.3**	4.5 ± 1.1	6.0 ± 1.1	8.4 ± 1.0	24.5 ± 2.9	75.6 ± 6.3	**317.3 **±** 32.0**
EV F1	17.3 ± 0.7	188.2 ± 6.6	174.8 ± 1.7	8.0 ± 0.6	11.2 ± 0.3	3.2 ± 0.3	17.2 ± 1.1	88.7 ± 5.0	508.6 ± 4.5
EV G2	17.2 ± 1.0	204.4 ± 18.5	188.7 ± 10.0	9.3 ± 01.0	11.6 ± 0.8	2.3 ± 0.5	17.4 ± 0.8	96.6 ± 3.3	547.6 ± 29.4

Average ± SEM of four independent cultures. Bold indicates a significant difference with both empty vector (EV) controls (ANOVA and Bonferroni's post hoc test, *P* < 0.05). Fatty acid descriptions as in Table 1.

## Discussion

### Expanding transgenic techniques in *N. oceanica*

A strong biotechnological interest has driven development of transgenic tools for *Nannochloropsis* species in recent years (Hu *et al*., 2014; Kang *et al*., 2015b; Kilian *et al*., 2011; Radakovits *et al*., 2012; Vieler *et al*., 2012). However, a comprehensive and modular protein production toolkit vector set allowing the engineering of convenient epitope‐tagged fusion proteins was lacking. Such a toolkit with multiple genetic reporters, selection markers, two additional promoters and several strategies for multigene expression is now available.

We have tested two new promoters for protein overproduction in *N. oceanica* CCMP1779. To enhance expression of single transgenes in *Nannochloropsis* species, engineers have used several endogenous promoters including the lipid droplet surface protein (LDSP) (Kaye *et al*., 2015; Vieler *et al*., 2012), β‐tubulin, heat‐shock protein 70 and ubiquitin extension promoters (UEP) (Kang *et al*., 2015a; Radakovits *et al*., 2012). We have tested an elongation factor promoter that displays high and stable expression throughout light:dark cycles (Poliner *et al*., 2015), and has been utilized in diverse organisms (Gill *et al*., 2001; Kim *et al*., 1990), including diatoms (Seo *et al*., 2015). A variety of promoters allow gene expression at different levels and in response to different environmental conditions. Furthermore, repeated use of transgenic elements can lead to genetic instability and as gene stacking techniques mature in *Nannochloropsis* species, multiple promoters are desirable to modulate the expression of many genes.

Bidirectional promoters are extremely useful for multigene expression. The endogenous bidirectional promoter (VCP2) between a pair of violaxanthin/chlorophyll a‐binding proteins (*NannoCCMP1779|4698* and *NannoCCMP1779|4699*) has been used to drive resistance genes (Kilian *et al*., 2011) and has been paired with coding sequences for fluorescent proteins with subcellular location tags (Moog *et al*., 2015). We have tested the ribosomal component S15 and S12 bidirectional promoter (Ribi) and identified several additional bidirectional promoters that may also be suitable for transgenic expression including histones, other VCPs and nitrate reductase (Table S3). These bidirectional promoters can be used for selection of high target transgene expression or for reducing potential silencing effects of transgenes by linking transgene production to a resistance marker.

Transgenic techniques in a number of eukaryotes have exploited 2A peptides, which enable a single transcript to encode multiple discrete protein products. A variety of 2A peptides have been identified and exploited with differing levels of efficiency in a number of eukaryotes, including yeast (Doronina *et al*. 2008), animals (Kim *et al*., 2011; Szymczak *et al*., 2004), plants (Burén *et al*., 2012), insects (Wang *et al*., 2015) and algae (Plucinak *et al*., 2015; Rasala *et al*., 2012). Applying this approach to stramenopiles, we have successfully developed an extended P2A sequence that is efficient for peptide bond skipping in *N. oceanica* CCMP1779. When placed between a resistance gene and firefly luciferase reporter, we obtained skipping efficiency of greater than >60%. Moreover, the overexpression of the *Δ9* FAD coding sequence downstream of BleR‐P2A(30) resulted in nonsignificant increase in 18:1^*Δ9*^, indicating enzymes produced as a P2A‐mediated fusion with a resistance gene are functional in *N. oceanica* CCMP1779. Our extensions of the P2A sequence showed diminishing increases in cleavage efficiency suggesting an optimal length has been found (Figure S4). Therefore, the P2A peptide is a promising tool for producing discrete proteins from a single reading frame in *N. oceanica* CCMP1779, and further mutational studies of the P2A sequences could potentially yield higher performing variants.

We anticipate that the development of the pNOC‐OX and pNOC‐stacked expression vectors will facilitate research in *Nannochloropsis* species and lay the foundation for combinatorial genetic engineering in an oleaginous microalga chassis for synthetic biology.

### Characterization of the EPA biosynthetic pathway

As an example for multigene metabolic engineering in *N. oceanica* CCMP1779, we identified and characterized the four fatty acid desaturases and one elongase involved in the production of the LC‐PUFA, EPA (20:5). Heterologous expression in *S. cerevisiae* confirmed the predicted biochemical function of each gene in the pathway, leading to EPA production without the supply of external fatty acids (Figure 3, Table S1). We also showed that the *Δ5* and ω*3* FADs can both use ω*3* or ω*6* fatty acids when reconstituted in yeast (Figure S1). Similarly, *N. oceanica Δ6* FAD expressed in *S. cerevisiae* was able to process ω*3* or ω*6* fatty acids when exogenously supplied (Ma *et al*., 2011), suggesting substrate preference may be determined by glycerolipid acyl carriers.

We used our gene stacking vector toolset (Figure 4) to increase expression levels of single or multiple endogenous genes involved in EPA synthesis. Strains overexpressing one, two or three FAD encoding genes displayed elevated fractions of LC‐PUFAs, notably EPA (Table 1). However, we did not observe a further increase in LC‐PUFAs in the lines overproducing more than one enzyme. The isolation of the EPA biosynthetic genes enables further studies into regulation of the pathway and provides tools useful for manipulation of LC‐PUFA production in *N. oceanica* and heterologous hosts.

### Metabolic engineering for increased EPA content in *N. oceanica*

The overexpression of single *Δ5* or *Δ12* FADs led to an approximate 25% increase in EPA mol ratio (Table 1), in line with observations in other stramenopiles. The overproduction of an endogenous *Δ5* FAD in *Phaeodactylum tricornutum* under the control of a fucoxanthin chlorophyll a/c binding protein gene promoter led to a 58% increase in EPA (Peng *et al*., 2014). Overproduction of an elongase involved in LC‐PUFA production in *P. pseudonana* led to a 40% increase in EPA (Cook and Hildebrand, 2015). It has been previously shown that in *N. oceanica* CCMP1779, the inducible overproduction of the *Δ12* FAD using the LDSP promoter produced a 50%–75% increase in the 20:4^*Δ5,Δ8,Δ11,Δ14 *^mol ratio during nitrogen deprivation and stationary phase (Kaye *et al*., 2015).

The FAD genes in *N. oceanica* CCMP1779 are coexpressed under diurnal light:dark cycles during exponential growth (Figure 1) (Poliner *et al*., 2015). Therefore, we had initially hypothesized that high expression of multiple enzymes is necessary to maintain flux through the pathway and further increase end‐product concentration. However, the overproduction of the multiple desaturase proteins in the DOX9 + 12 and DOX5 + 9 + 12 lines did not have an additive effect. These results suggest that there might be a limit to PUFA content in *N. oceanica* CCMP1779 before cell physiology is affected under the growth conditions tested.

The cell division rate of DOX lines was not reduced when compared to wild‐type cells. However, decreases in total cellular fatty acids of DOX lines expressing the *Δ12* or *Δ5* FAD coding sequences indicates that the physiology of *N. oceanica* is compromised by desaturase overproduction (Table 2). In *N. oceanica,* EPA is an endogenous major LC‐PUFA that is associated with polar lipids of plastidic membranes and likely has a functional role in the photosynthetic membrane (Valentine and Valentine, 2004). Typically, in response to environmental conditions, membrane saturation is altered to maintain membrane fluidity, and LC‐PUFAs are increased in membranes during lower temperatures and high light. Interference with native membrane composition may have deleterious effects that limit the accumulation of EPA beyond certain levels.

A higher relative increase in some LC‐PUFAs has been achieved when the strategy has been to introduce novel LC‐PUFAs (Ruiz‐Lopez *et al*., 2014; Xue *et al*., 2013) or to elevate the level of minor LC‐PUFAs, initially present in small amounts (Hamilton *et al*., 2014). For example, the heterologous expression of both a *Δ6* FAD and a *Δ5* FAE coding sequence in *P. tricornutum* led to a stronger DHA accumulation than in the single overexpressing lines (Hamilton *et al*., 2014). However, wild‐type *P. tricornutum* contains only trace amounts of DHA and in these transgenic lines, the accumulation of DHA correlated with a strong decrease in EPA levels, indicating that these transgenics increased partitioning towards DHA but not an overall increase in flux through the LC‐PUFA pathway. These observations support the hypothesis of a limit of LC‐PUFA imposed on the cell under specific growth conditions.

In addition to possible negative effects on algal physiology, it is likely that other steps in LC‐PUFA biosynthesis, such as fatty acid elongation or *Δ6* or ω*3* desaturation, are rate limiting and need further enhancement to increase LC‐PUFA content. Moreover, it is possible that increased LC‐PUFA turnover by β‐oxidation is involved in maintaining a balanced LC‐PUFAs content (Moire, 2004; Xue *et al*., 2013). Down‐regulation of the native EPA biosynthetic pathway at a transcriptional or post‐transcriptional level may also compensate for the overproduction of FADs described here.

Strategies for sequestration of LC‐PUFAs in TAG could be an option to overproduce LC‐PUFAs without compromising cell physiology (Kaye *et al*., 2015). Although *N. oceanica* contains little LC‐PUFAs in TAG under normal conditions, the content of LC‐PUFAs in TAG increases following cellular stresses (Liu *et al*., 2013; Vieler *et al*., 2012). We observed a decrease 16:0 and 16:1 per cell in the DOX lines (Table 2); however, the overexpression of a *Δ12* FAD using a stress‐inducible promoter (Kaye *et al*., 2015) did not cause such a decrease, indicating that the timing of gene expression is likely to be important for minimizing negative effects of enhanced LC‐PUFA content by maybe sequestering LC‐PUFAs in TAG. Diacylglycerol acyltransferases (DGATs) and phosphatidylglycerol acyltransferase (PDATs) that have preferences for 20C fatty acids also offer potential tools for channelling EPA to the TAG stores (Manandhar‐Shrestha and Hildebrand, 2015; Xu *et al*., 2013). *N. oceanica* contains 12 DGATS and two PDATs that are likely to have different substrate preferences, including for LC‐PUFAs (Zienkiewicz *et al*., 2017). Studies into the functional effects of altering the fatty acid profile as well as identifying compensating internal forms of regulation are needed to identify strategies for further accumulation of LC‐PUFAs.

## Experimental procedures

### Growth conditions

Axenic cultures were grown in shaking flasks of F/2 medium under 100 μm/s/m^2^ white light, at 22 °C and 120 rpm. For protein, metabolite and gene expression analyses, cells were grown under constant light and samples were collected from mid‐log cultures (30 × 10^6^ cells/mL).

### Cloning of *N. oceanica* CCMP1779 EPA pathway genes

Axenic cultures were under 12:12 light:dark cycle at 22 °C (Poliner *et al*., 2015). Cell counts and cell size measurements were obtained using a Coulter Counter Z2 (Beckman Coulter) using a profile with a range of 1.8–3.6 μm. *N. oceanica* CCMP1779 at mid‐log phase was used for RNA isolation as described previously (Poliner *et al*., 2015). First‐strand DNA synthesis was accomplished using SuperScript III with oligo dT(NEB). cDNAs were amplified using primers shown in Table S4 and Q5 polymerase (NEB), blunt cloned into pCR‐Blunt (Thermo Scientific) and sequenced.

### Yeast transformation and expression

EPA pathway genes were cloned into yeast expression vectors containing galactose‐inducible promoters. The elongase PCR product was integrated into pYES2.1‐topo (Invitrogen). Desaturases were amplified with the addition of C‐terminal 6X histidine tails and restriction sites for integration into pESC‐his and pESC‐leu (Agilent). The PCR product was digested with the noted restriction enzymes (Table S4) and ligated into the yeast expression vectors. InvSc1 yeast cells (Kajiwara *et al*., 1996) were transformed with the expression vectors using the Frozen‐EZ Yeast Transformation II Kit (Zymo Research) and selected on SC (ClonTech) medium with proper dropout auxotrophy selection. Several colonies from each transformation were selected for further experimentation and were grown in 5 mL of SC overnight at 30 °C. The overnight cultures were collected by centrifugation at 1000 ***g*** for 5 min, thoroughly decanted, resuspended in 5 mL of SC without sugar, histidine, leucine and uracil and OD600 was measured in duplicate. For the zero‐hour time point fatty acid analysis, 0.5 mL culture was collected by centrifugation at 13 000 ***g***, decanted and frozen in liquid nitrogen. Flasks of 5 mL SC 2% galactose were inoculated at 0.4 OD600 and grown at 20 °C, and 24‐h and 48‐h time points were collected. For substrate feeding, 0.1% NP‐40 (Sigma‐Aldrich), as a detergent, and 0.5 mm free fatty acids (Santa Cruz Biotechnology) in glucose and galactose SC medium were included. Fatty acid analysis with washed cell pellets and 5 μg of pentadecanoic acid internal standard were conducted. LC‐PUFA authentic standards were prepared in a separate reaction for confirmation of LC‐PUFA running times. Fatty acid methyl ester preparation and extraction were performed as described previously (Liu *et al*., 2013).

### Protein sequence analysis

Protein sequences were generated by translation of the obtained cDNA using canonical codon usage. To identify functional domains, the protein sequences were submitted to CDD BLAST (http://www.ncbi.nlm.nih.gov/Structure/cdd/wrpsb.cgi). To identify localization signals, protein sequences were submitted to HECTAR (http://webtools.sb-roscoff.fr/) (Gschloessl *et al*., 2008). To identify transmembrane domains, protein sequences were submitted to TMHMM2 (http://www.cbs.dtu.dk/services/TMHMM/).

### Identification of bidirectional promoters in *N. oceanica* CCMP1779

A custom python script was used to assemble the coding regions of each gene as determined in the genome assembly and annotation of *N. oceanica* CCMP1779 (Vieler *et al*., 2012), and only genes with start and stop codons were added to the final list. These coding regions were assessed with the CUSP function of the EMBOSS program (Table S2). A custom python script was used to identify diverging gene pairs with intergenic regions of <1500 base pairs, with suitable gene expression during light:dark cycles (Poliner *et al*., 2015) (Data S1). Putative gene pairs were manually examined to determine functional annotation (Table S3).

### Construction of *Nannochloropsis* expression vectors

All constructs were derived from the pNoc‐Dlux vector (Data S2), a pGEM‐derived plasmid containing a hygromycin resistance cassette (Vieler *et al*., 2012) and a gateway–firefly luciferase cassette. All of the vectors for expression in *S. cerevisiae* and *N. oceanica* are listed in Table S5, and the complete annotated sequences are included in Data S2.

### *Nannochloropsis* transformation

Vectors were linearized by restriction digestion, and purified and concentrated by ethanol precipitation. *N. oceanica CCMP1779* transformation was performed according to the method of Vieler *et al*. (2012) with 3 μg of vector DNA, with 30 μg carrier DNA (Invitrogen UltraPure™ Salmon Sperm DNA Solution). Transformed cells were allowed to recover for 48 h and then plated in top agar with the respective selection. After 3–4 weeks, individual colonies were resuspended in 100 μL F/2. From each transformation, ~20 colonies were screened for increased LC‐PUFA content, and two to three colonies identified as positive.

### *Nannochloropsis* luminescence assays

*N. oceanica* CCMP1779 culture was mixed with F/2 supplemented with either firefly luciferin (Gold Biotech) or NanoLuciferase substrate (Promega), at a final volume of 200 μL, with 500 μm firefly luciferin or 10 000 ×  dilutions of NanoLuciferase substrate. For normalized measurements, 1 million *N. oceanica* cells were used. Luminescence was measured with a Centro XS3 LB960 luminometer (Berthold Technologies) over a 0.3‐s exposure.

### Expression analysis in *N. oceanica* CCMP1779

For protein expression, frozen pellets from 5 mL culture were ball‐milled in 2‐mL tubes (30 Hz, 2 min) with a TissueLyser II (Qiagen). After addition of protein extraction buffer (100 mm Tris pH 8.0, 2 mm PMSF, 2% B‐mercaptoethanol, 4% SDS), the sample was heated to for 3 min (60 °C for FADs, 80 °C for other proteins), centrifuged at 13,000 ***g*** for 3 min and the supernatant was transferred to new tube. Protein content was determined using the RCDC assay (Bio‐Rad), and equal quantities of protein were loaded for SDS‐PAGE. Proteins were transferred to PVDF membranes (Bio‐Rad) overnight at 4 °C. Blots were blocked in TBST with 5% milk for 1 h at room temperature and washed six times with TBST. For GFP detection, we used α‐GFP antibody (Abcam ab5450) 1:1000 in TBST with 5% BSA for 1 h, and a secondary donkey α‐goat HRP antibody (Santa Cruz sc‐2020) 1:10 000 in TBST with 5% milk. For HA detection, we used α‐HA‐HRP antibody solution (Roche 3F10) at 1:1000 in TBST with 5% milk for 1 h. Signals were detected using clarity chemiluminescence reagent (Bio‐Rad). Band quantification was conducted using Image Lab software (Bio‐Rad).

RNA isolation, cDNA synthesis and real‐time PCR were performed as described previously (Poliner *et al*., 2015). Real‐time PCR primers were checked for efficiency and specificity. The delta‐delta Ct method was used to determine gene expression relative to the gene encoding the actin‐related protein (*ACTR*) *NannoCCMP1779_1845*.

### Fatty acid methyl ester extractions in *N. oceanica*

Cells (2 mL) were collected by filtration through GF/C filters, and filters were stored in screw top tubes at −80 °C. Fatty acid methyl ester (FAME) extraction and analysis were carried out as described previously (Liu *et al*., 2013).

### Confocal microscopy

Cerulean detection in transformed *N. oceanica* CCMP1779 was carried out with Olympus Spectral FV1000 microscope (Olympus, Japan) at the excitation wavelength of 435 nm (a diode laser). For endoplasmic reticulum labelling, 50 nm DiOC6 (Sigma‐Aldrich) in F/2 medium was used. Cells were labelled directly before microscopic analysis. An argon (488 nm) laser was used for DiOC6 excitation. Chloroplast autofluorescence was excited using a solid‐state (515 nm) laser. CLSM figures represent Z‐series images composed using the Olympus FluoView FV1000 confocal microscope software (Olympus).

## Supporting information


**Figure S1** The final two steps of EPA biosynthesis in *S. cerevisae* with exogenous supply of substrates.
**Figure S2** Modification of the Ribi promoter to remove restriction sites.
**Figure S3** Assessment *N. oceanica* CCMP1779 promoters’ strength using Nano‐luciferase.
**Figure S4** N‐terminal extended 2A peptide screening for increased ribosomal skipping efficiency.
**Figure S5** CLSM analysis of *N. oceanica* CCMP1779 wild‐type, and empty vector and CFP‐desaturase overexpressing (DOX) transformants.

**Table S1** Fatty acid mole percentage of *S. cerevisiae* strains.
**Table S2** Codon usage of *N. oceanica* CCMP1779.
**Table S3** Bidirectional gene pairs.
**Table S4** Primers used in study.
**Table S5** List of constructs generated in this study.

**Data S1** Script used for the identification of bidirectional promoters.

**Data S2** Description of vector construction and sequences of vectors generated in this study.
