# Supplementary material for: A toolkit for Nannochloropsis oceanica CCMP1779 enables gene stacking and genetic engineering of the eicosapentaenoic acid pathway for enhanced long‐chain polyunsaturated fatty acid production
Source: Plant Biotechnol J. 2017 Jul 13;16(1):298–309. doi: 10.1111/pbi.12772 (PMC5785352; doi:10.1111/pbi.12772)
Supplement: Supplementary file 1 — Figure S1 The final two steps of EPA biosynthesis in S. cerevisae with exogenous supply of substrates. Figure S2 Modification of the Ribi promoter to remove restriction sites. Figure S3 Assessment N. oceanica CCMP1779 promoters’ strength using Nano‐luciferase. Figure S4 N‐terminal extended 2A peptide screening for increased ribosomal skipping efficiency. Figure S5 CLSM analysis of N. oceanica CCMP1779 wild‐type, and empty vector and CFP‐desaturase overexpressing (DOX) transformants. [file PBI-16-298-s002.pdf]

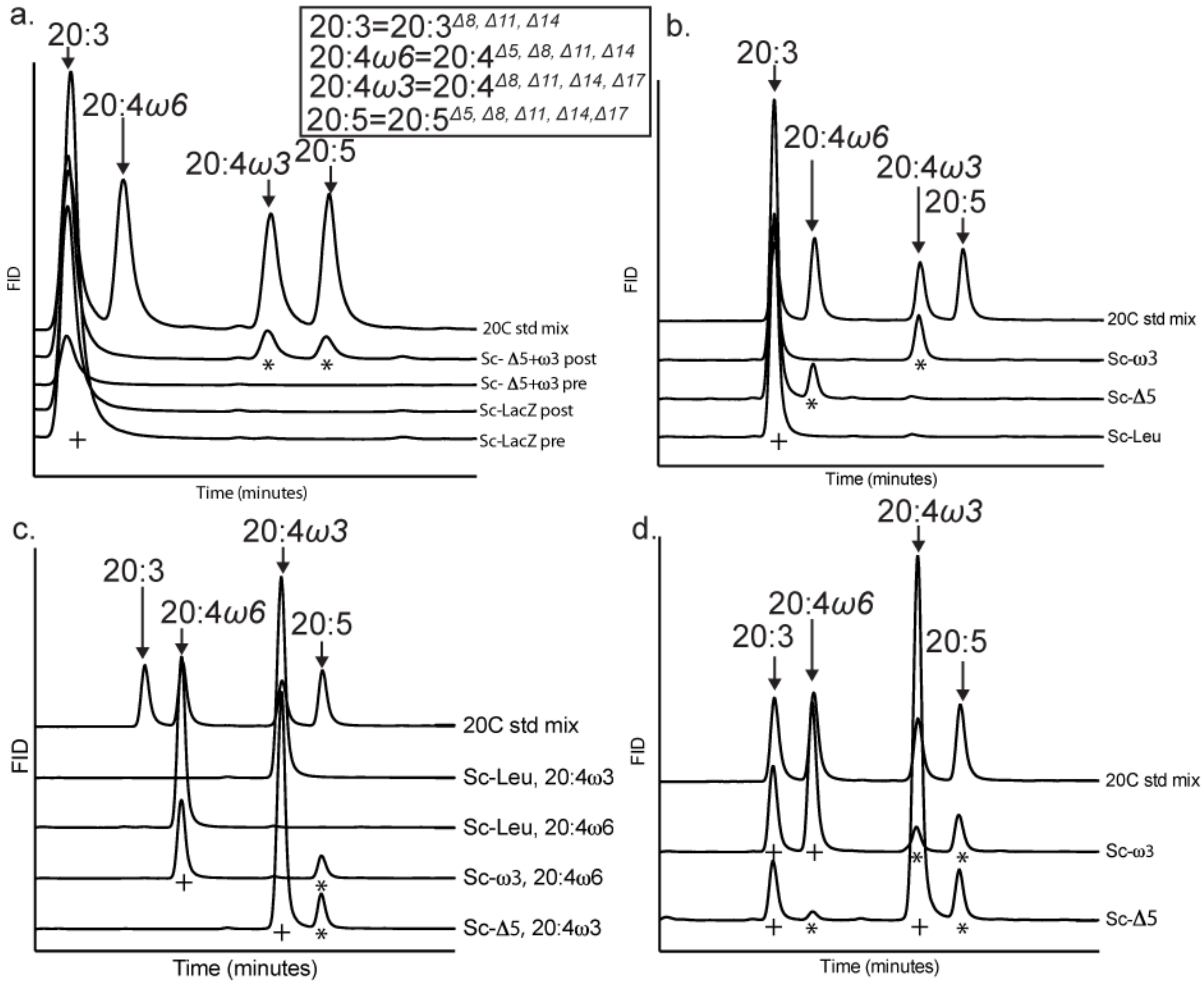

Figure S1. Functional characterization of EPA biosynthesis enzymes in *S. cerevisae* (Sc). Representative GC-FID fatty acid profiles. In the chromatograms, fatty acids supplied in the media are indicated with a (+), and fatty acids produced by yeast are indicated with a (*) underneath the respective peaks. 20C std mix, indicates standards of 20 carbon LC-PUFAs. (a) Yeast exogenously supplied with $20{:}3^{\Delta8,\Delta11,\Delta14}$ and expressing either FAD Δ5 and ω3 (Sc-Δ5+ω3), or the LacZ control gene (Sc-LacZ) before (pre) or after (post) 48 h gene expression induction by galactose. (b) Yeast exogenously supplied with $20{:}3^{\Delta8,\Delta11,\Delta14}$ and expressing either FAD ω3 (Sc-ω3), FAD Δ5 (Sc-Δ5), or the Leu control gene (Sc-Leu) after 48 h gene expression induction by galactose. (c) Yeast exogenously supplied with either 20:4*ω3* ($20{:}4^{\Delta8,\Delta11,\Delta14,\Delta17}$) or 20:4*ω6* ($20{:}4^{\Delta5,\Delta8,\Delta11,\Delta14}$), and expressing either FAD ω3 (Sc-ω3), FAD Δ5 (Sc-Δ5), or the Leu control gene (Sc-Leu) after 48 h gene expression induction by galactose. (d) Yeast expressing

either FAD ω3 (Sc-ω3) or FAD Δ5 (Sc-Δ5) and exogenously supplied with 20:4*ω3* ($20{:}4^{\Delta 8,\Delta 11,\Delta 14,\Delta 17}$) and 20:4*ω6* ($20{:}4^{\Delta 5,\Delta 8,\Delta 11,\Delta 14}$) respectively after 48 h gene expression induction by galactose.

a. >nanno_989:5127..6310

tctgtggatggagggagggaaggagggggggagtgagtgagtcgagagaacgacgactggggcaaagagaggatgacgccactgcaacaaaa
gacagggaagaatagaggctgttggaaaacaggaacacagtgaagaatagaggctgcagggtaacaggcacatttatcgcagagaggcgggcg
gcaggggcgatacgtaaagagacccctaaacttctccgcgtcatccgttcacgctacctccttccctccctttactcccattgatcaccaaggcagcgc
ccgcagccaccagcagccaccgtcctcagtgtcacagctag**acgcgt**ccccatcaggcagcagaccaccagaatatgcgctttcgcttcccatcat
atccctccctcctccccttctattcctcatcatgcatcatttctttctccttgctgcatgcccctcttcttaccttggctaagatctaccggaggaagaagtctt
caacgatagataaatgcctgtatcgtgatggttgtttgaatggtggaagtgagccaggtattgagaacgaggggcgcaagggtgcgtggaagaagg
cagggagaggcgaggcgaaggagaagggcatgtggtgggaacgaagcctccacaccacagccacccatgcttttctctgttcgagttcccgggtt
ctactctcacacactacacgtgcacagaaaacattcacgcacaacagagatacagagggatccgtcgagatggtctacacgtcctaatgtcccctgct
ttcgaagcccagagccatcgctttctgtttttatccattcgcaacacatcctgttc**tcgcga**cctctcctcttcctggccaccatcgcttctccgtgccatgc
cctgtacatccttccctccctcgttgtctaccacactaatcatgcgctctgcagcagacactcggctactatttgtgtcgtacggattgaggggagggag
gggaagggaggcaagaagtaaatcggtggtcaacaacagacgcggtctccccaccctccctcattcctttcccctcctataatgagcgaagaaagcc
atggacaccgagtcacccactctcctaatccaccaccaccagggcactaacggactcacgctcaccaaactcggtaacaatatccactgcacacaga
tacacacgcaacc

b. >Ribi sdm

tctgtggatggagggagggaaggagggggggagtgagtgagtcgagagaacgacgactggggcaaagagaggatgacgccactgcaacaaaa
gacagggaagaatagaggctgttggaaaacaggaacacagtgaagaatagaggctgcagggtaacaggcacatttatcgcagagaggcgggcg
gcaggggcgatacgtaaagagacccctaaacttctccgcgtcatccgttcacgctacctccttccctccctttactcccattgatcaccaaggcagcgc
ccgcagccaccagcagccaccgtcctcagtgtcacagctag**ac...t**ccccatcaggcagcagaccaccagaatatgcgctttcgcttcccatcatat
ccctccctcctccccttctattcctcatcatgcatcatttctttctccttgctgcatgcccctcttcttaccttggctaagatctaccggaggaagaagtcttca
acgatagataaatgcctgtatcgtgatggttgtttgaatggtggaagtgagccaggtattgagaacgaggggcgcaagggtgcgtggaagaaggca
gggagaggcgaggcgaaggagaagggcatgtggtgggaacgaagcctccacaccacagccacccatgcttttctctgttcgagttcccgggttcta
ctctcacacactacacgtgcacagaaaacattcacgcacaacagagatacagagggatccgtcgagatggtctacacgtcctaatgtcccctgctttc
gaagcccagagccatcgctttctgtttttatccattcgcaacacatcctgttc**tcAcga**cctctcctcttcctggccaccatcgcttctccgtgccatgccc
tgtacatccttccctccctcgttgtctaccacactaatcatgcgctctgcagcagacactcggctactatttgtgtcgtacggattgaggggagggaggg
gaagggaggcaagaagtaaatcggtggtcaacaacagacgcggtctccccaccctccctcattcctttcccctcctataatgagcgaagaaagccat
ggacaccgagtcacccactctcctaatccaccaccaccagggcactaacggactcacgctcaccaaactcggtaacaatatccactgcacacagata
cacacgcaacc

Figure S2. Modification of the Ribi promoter to remove restriction sites. (a) The genomic sequence of the intergenic region between the bidirectional gene pair, *NannoCCMP1779_9669* and *NannoCCMP1779_9669*, is shown and the restriction sites MluI and NruI are bolded and underlined. (b) The Ribi promoter after site directed mutagenesis. The modified restriction sites are bolded and underlined, with nucleotide deletions represented by periods and alterations shown in upper case.

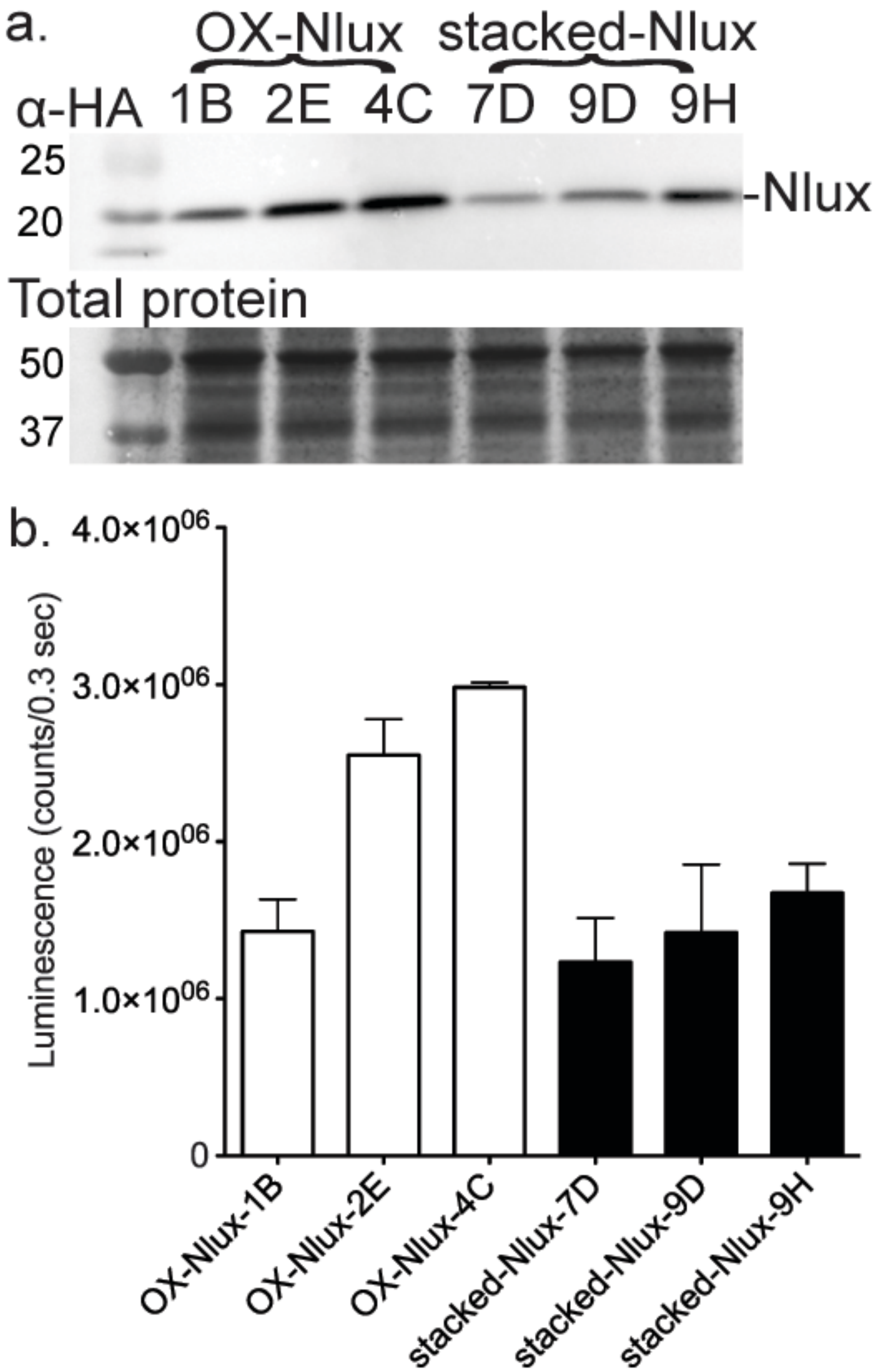

Figure S3. Assessment *N. oceanica* promoters' strength using Nano-luciferase. (a) Immunoblotting with α-HA of *N. oceanica* lines transformed with pNOC-OX-Nlux and pNOC-Stacked-Nlux detects Nlux-HA protein. Total protein stained using the dye DB71. (b) Normalized luminescence signal of *N. oceanica* lines transformed with pNOC-OX-Nlux and pNOC-Stacked-Nlux (average ± range, 2 technical replicates).

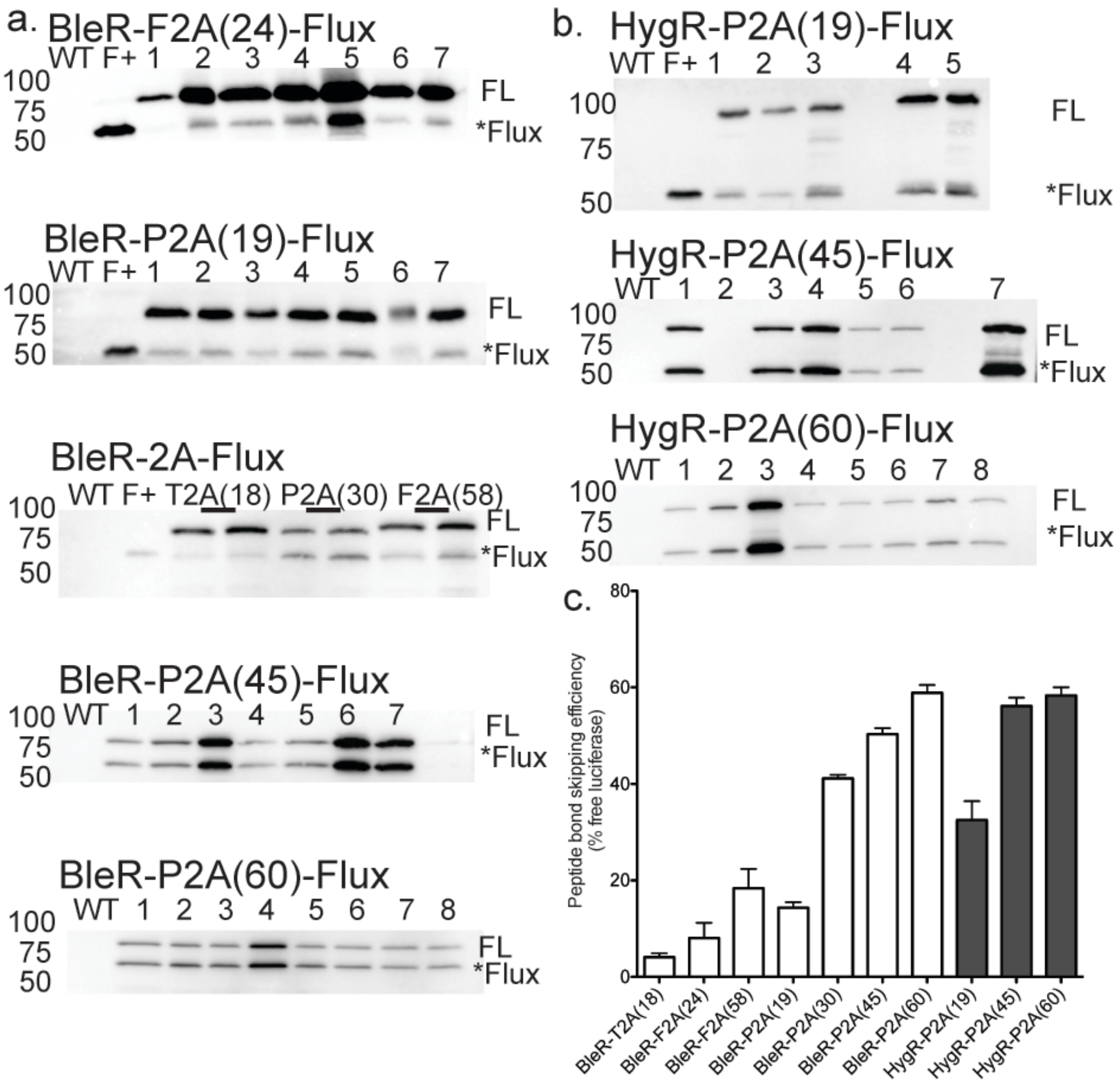

Figure S4. N-terminal extended 2A peptide screening for increased ribosomal skipping efficiency. (a) Numbers in parentheses correspond to numbers of amino acids of the 2A peptide. Immunoblotting with α-HA detects full-length (FL) and released firefly luciferase (*Flux). Assesment of transformants producing BleR linked to Flux by a variety of 2A peptides: BleR-F2A(24)-Flux, BleR-P2A(19)-Flux, BleR-T2A(18)-Flux, BleR-P2A(30)-Flux, BleR-F2A(58)-Flux, BleR-P2A(45)-Flux, and BleR-P2A(60)-Flux. (b) Assesment of transformants producing HygR linked to Flux by different length P2A peptides: HygR-P2A(19)-Flux, HygR-P2A(45)-Flux, and HygR-P2A(60)-Flux. (c) Peptide bond skipping quantified using densitometric measurements of FL and Flux quantities and efficiency determined with the equation (*Flux/(FL+*Flux))×100; (average ± SEM, n=2-8).

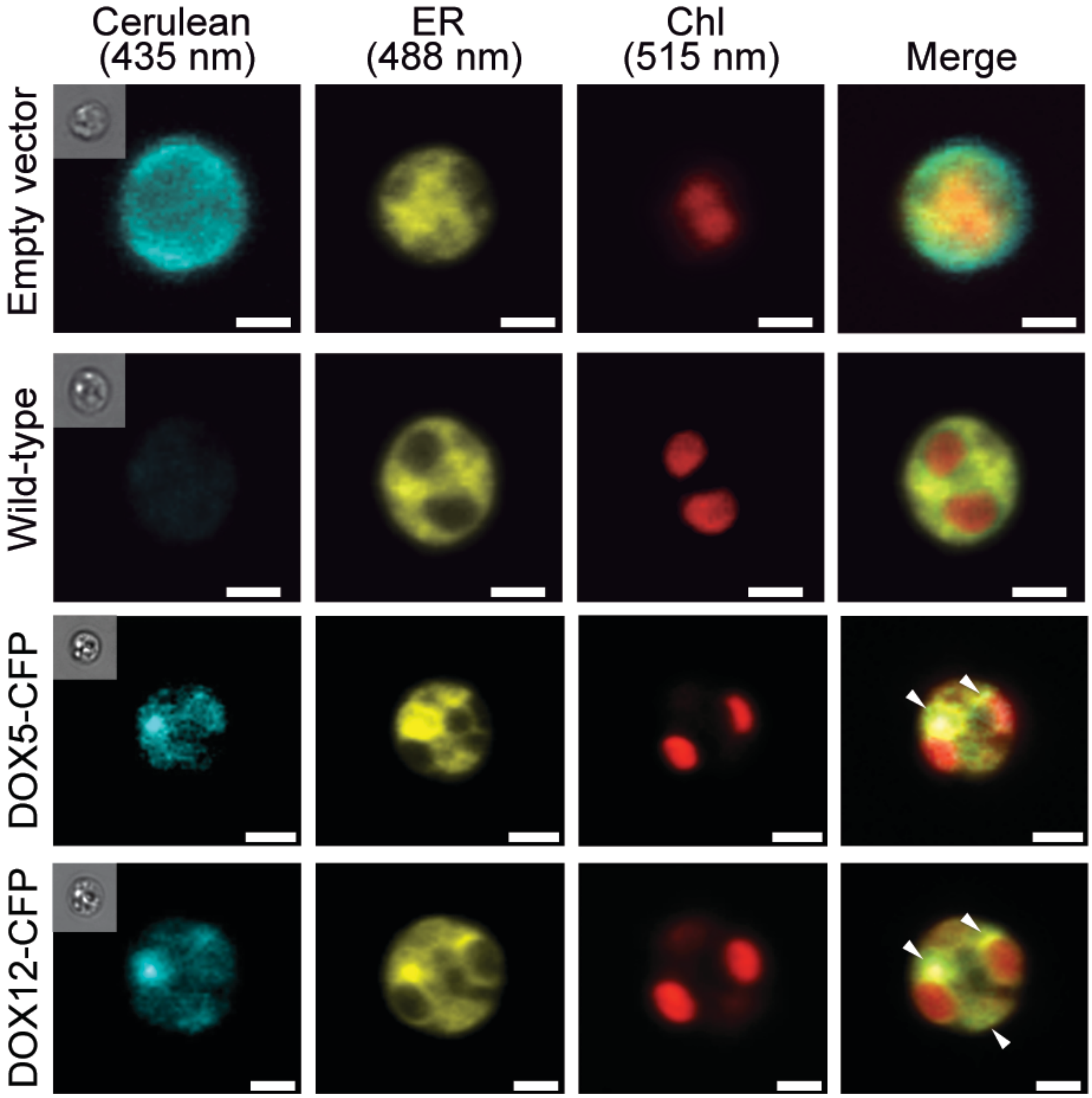

Figure S5. CLSM analysis of *N. oceanica* CCMP1779 wild type, and empty vector and CFP-desaturase overexpressing (DOX) transformants. Cells were examined for the presence of cerulean fluorescence. Staining with an ER specific fluorescent dye and chlorophyll autofluorescence were compared to the cerulean signal. Arrowheads indicate co-localization of ER and cerulean fluorescence. Bars = 1.5 μm.

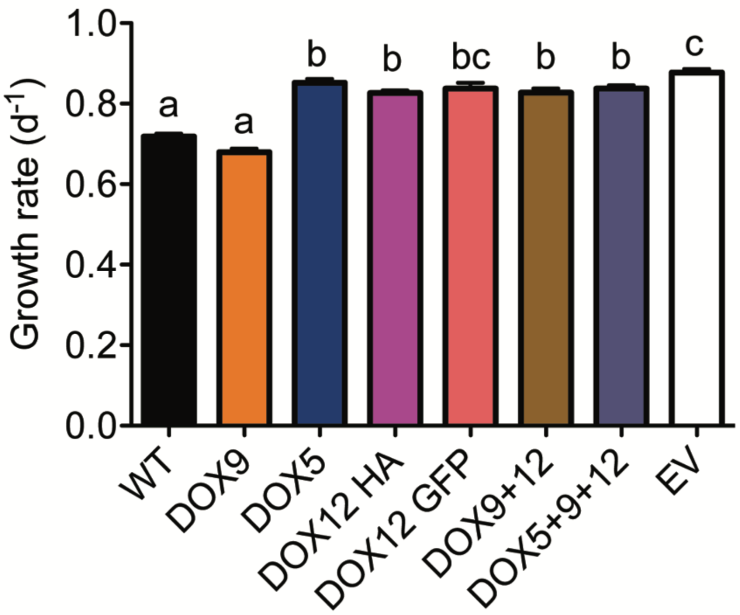

Figure S6. Growth rates of *N. oceanica* CCMP1779 DOX lines during exponential growth. Growth rates were estimated using the first four days of the growth curves shown in Figure 7b (average ± SEM, 8-12 independent cultures from 2-3 lines). Cell counts were natural log transformed and growth rates calculated using linear regression. Values statistically different are labeled with different letters ($p<0.05$; ANOVA followed by Bonferroni's post hoc test).
